# Coordinating Earthquake Response in Neonatal Intensive Care: A Phenomenological Exploration of Nurses' Experiences

**DOI:** 10.1111/nhs.70394

**Published:** 2026-07-18

**Authors:** Birgül Erdoğan, Sümeyra Topal, Sinem Yalnızoğlu Çaka

**Affiliations:** ^1^ Department of Pediatric Nursing, Faculty of Health Science Kocaeli University Kocaeli Turkey; ^2^ Department of Pediatric Nursing, Gülhane Faculty of Nursing Health Sciences University Ankara Turkey

**Keywords:** disaster preparedness, disaster response, earthquake, neonatal intensive care unit, neonatal nurses, technology‐dependent care

## Abstract

Disaster response in neonatal intensive care units is particularly complex because care continuity depends on coordinated teamwork, stable infrastructure, and technology‐dependent support for highly vulnerable infants. However, qualitative evidence on how nurses experience disaster response in these settings remains limited, especially in the context of the 2023 Türkiye–Syria earthquakes. This study explored how nurses working in neonatal intensive care units experienced disaster response during the earthquakes. Data were collected through semistructured in‐depth interviews with 21 nurses and analyzed using descriptive phenomenological analysis. Participants described disaster response not only as a clinical emergency but also as a disruption of the systems supporting coordination, safe neonatal care, and practical preparedness. They reported breakdowns in communication and role clarity, fragility in care when electricity, oxygen delivery, monitoring systems, evacuation planning, and essential supplies became unstable, and a clear gap between general disaster education and unit‐specific readiness. Overall, the findings highlight the need for neonatal intensive care‐specific disaster preparedness.

## Key Points

- Disaster response in neonatal intensive care units was shaped not only by clinical urgency but also by the breakdown of routine coordination, communication, and role clarity.
- The fragility of technology‐dependent neonatal care became particularly visible when electricity, oxygen delivery, monitoring systems, evacuation pathways, and essential supplies were disrupted.
- Neonatal intensive care nurses described a gap between general disaster education and the practical, unit‐specific preparedness required during a real earthquake, highlighting the need for regular simulation‐based training and neonatal intensive care‐specific disaster planning.

## 1 Introduction

Natural disasters, particularly large‐scale earthquakes, place substantial pressure on healthcare systems by disrupting service delivery, communication, infrastructure, staffing, and access to essential resources. Under such conditions, continuity of care depends not only on the physical resilience of health facilities but also on the preparedness, coordination, and responsiveness of healthcare professionals (World Health Organization 2024; Kiymis and Yuce 2025). Nurses are central to this response because they must sustain patient care while simultaneously managing uncertainty, rapid decision‐making, and team‐based action in unstable and resource‐constrained environments (Annak Mercan et al. 2025; Gunawan et al. 2019).

Neonatal intensive care units (NICUs) are among the most vulnerable hospital settings during disasters because they provide care for critically ill and highly dependent infants whose survival often relies on continuous monitoring, respiratory support, thermal regulation, medication management, and technology‐based care (Lin et al. 2015; Eskandar‐Afshari et al. 2020). In these settings, even brief interruptions in electricity, oxygen delivery, communication systems, or equipment functionality may have immediate and serious consequences for patient safety. In addition to routine clinical care, NICU nurses may therefore be required to manage emergency evacuation, prioritization of infants, limited equipment and supply use, coordination with multidisciplinary teams, and communication with families under severe time pressure.

Existing literature has identified several factors that weaken disaster preparedness in critical care settings, including inadequate infrastructure, impractical disaster plans, communication breakdowns, shortages of equipment and supplies, and training that remains largely theoretical rather than practice‐based (Ma et al. 2020; Mousavipour and Sohrabizadeh 2021; Kuppanda et al. 2021). These limitations may be especially consequential in NICUs, where disaster response must account for the exceptional fragility of neonates and the technology‐dependent nature of care. Nevertheless, research focusing specifically on NICU nurses' disaster experiences remains limited, and qualitative evidence exploring how nurses experience and make sense of disaster response in these settings is still scarce.

This gap is particularly evident in the Turkish context. Türkiye is a country with high earthquake risk, yet disaster preparedness policies and operational guidance tailored to neonatal intensive care remain limited (Karaman et al. 2024; Topal et al. 2024). The 2023 Türkiye–Syria earthquakes exposed major challenges across healthcare services in the affected region, including infrastructure disruption, communication failures, workforce shortages, evacuation difficulties, and the urgent need to sustain safe care under crisis conditions (Annak Mercan et al. 2025; Topal et al. 2024). For NICU nurses, these challenges unfolded in a uniquely demanding environment in which maintaining respiration, monitoring, thermoregulation, and safe transfer of fragile infants became immediate priorities (Barfield and Krug 2017; Eskandar‐Afshari et al. 2020; Mousavipour and Sohrabizadeh 2021; Topal et al. 2025).

Although postdisaster reports and broader disaster nursing studies have highlighted the importance of communication, teamwork, leadership, and training, less is known about how these issues were experienced within neonatal intensive care environments during the early phase of a major earthquake response (Almukhlifi et al. 2021; Annak Mercan et al. 2025; Sellers et al. 2022). Understanding these experiences is important because it can provide context‐specific insight into the organizational, infrastructural, and educational needs that shape disaster preparedness in NICUs.

The aim of this study was to explore the experiences of NICU nurses during the 2023 Türkiye–Syria earthquakes, with particular attention to disaster preparedness, team coordination, infrastructure‐related challenges, and training needs in the early phase of disaster response.

## 2 Materials and Methods

### 2.1 Qualitative Approach

This study adopted a qualitative phenomenological approach to explore how NICU nurses experienced and made sense of disaster response during the 2023 Türkiye–Syria earthquakes. A phenomenological design was chosen because the study aimed to examine the meaning and essence of nurses' lived experiences in a highly stressful, technology‐dependent, and life‐threatening clinical context, rather than to identify the frequency of predefined problems (Creswell and Poth 2016). Data were collected through individual semistructured, in‐depth interviews, which enabled participants to describe their experiences in detail and allowed the researcher to further explore experience‐based meanings. The study was reported in accordance with the Consolidated Criteria for Reporting Qualitative Research (COREQ), a checklist developed to support transparent reporting of interview‐based qualitative studies (Tong et al. 2007).

### 2.2 Context

The study was conducted with nurses working in NICUs in provinces across southern and central Türkiye that were affected by the 2023 Türkiye–Syria earthquakes. These regions experienced major disruptions in health service delivery, infrastructure, communication, and access to essential resources during the early phase of the disaster response. The study focused on nurses' experiences during the first 24 h of the disaster response, a period marked by acute uncertainty, disrupted infrastructure, communication difficulties, and urgent neonatal care needs. Data were collected retrospectively between July and August 2025 to capture participants' accounts of working in a highly vulnerable, technology‐dependent care environment during the early phase of the disaster response.

### 2.3 Sampling Strategy and Participants

A snowball sampling strategy was used to recruit nurses who had direct experience of working in a NICU during the 2023 Türkiye–Syria earthquake response. This strategy was considered appropriate because the study aimed to reach participants with first‐hand experience of the phenomenon and because the target group was geographically dispersed across earthquake‐affected provinces. Initial participants who met the inclusion criteria were identified by the researchers and were subsequently asked to refer other eligible nurses. The inclusion criteria were being a registered nurse, having worked in a NICU in an earthquake‐affected province during the 2023 earthquakes, and being willing to participate in an online interview. A total of 21 nurses participated in the study. Recruitment continued until sufficient depth and richness of experiential accounts had been achieved and no substantially new meanings relevant to the study aim were emerging during the analytic process.

### 2.4 Data Collection

Data were collected through individual semistructured, in‐depth interviews conducted via Zoom between July and August 2025. Online interviewing was considered appropriate because participants were geographically dispersed across earthquake‐affected provinces and had varying work schedules. Before each interview, participants were informed about the purpose of the study, the voluntary nature of participation, confidentiality procedures, and the use of audio recording. Written and verbal informed consent were obtained prior to data collection. All interviews were conducted in Turkish, the native language of the participants and the interviewer. Considering the potentially distressing nature of recalling earthquake‐related experiences, participants were reminded before and during the interviews that they could pause or stop the interview at any time, decline to answer any question, or withdraw from the study without providing a reason. Each interview lasted approximately 45–60 min and was conducted by the same researcher. Open‐ended questions and follow‐up probes were used to elicit detailed accounts of participants' disaster preparedness, experiences during the earthquake, postdisaster practices, and recommendations for future improvement.

### 2.5 Data Collection Instruments

Data were collected using a participant information form and a semistructured interview guide. The participant information form included questions on age, educational status, and professional experience. The semistructured interview guide was developed based on the relevant literature and the research team's expertise in pediatric nursing and qualitative research. It was further refined following feedback from five experts, including four academics in pediatric nursing and one qualitative research specialist. The final interview guide comprised open‐ended questions addressing pre‐earthquake preparedness, experiences during the earthquake, postdisaster practices, and recommendations for improving future disaster preparedness. The main interview domains are presented in Table 1.

**TABLE 1:** Main interview domains and example questions for neonatal intensive care unit nurses.

Main interview domain	Example interview questions
Team coordination and intra‐team communication during the earthquake	How did you experience communication and coordination within the neonatal intensive care unit team during the earthquake? Were roles and responsibilities clearly defined within the team? How did teamwork and support mechanisms function during the disaster? What challenges did you face in following verbal orders and making rapid decisions? How did you cope with communication breakdowns during the earthquake?
Preparedness needs for neonatal intensive care units during earthquakes	Were there disaster preparedness plans in your unit prior to the earthquake? What were their limitations? Were safe evacuation and escape routes clearly established? Were critical materials and medical supplies, such as portable incubators, generators, and medications, sufficient? Were life‐support devices and methods adequate during postearthquake care?
Training and preparedness needs of nurses	Did you participate in disaster training and drills before the earthquake? Were they sufficient? Were unit‐specific practical trainings conducted? What shortcomings did you observe? Were postearthquake trainings or evaluations carried out in your institution? What strategies do you think could improve nurses' competence in disaster preparedness?

### 2.6 Data Processing

All interviews were audio‐recorded with participants' permission and transcribed verbatim by the researcher who conducted the interviews. The transcripts were checked against the audio recordings for accuracy and anonymized prior to analysis. Each participant was assigned an identifier code (e.g., P1, P2). Audio recordings, anonymized transcripts, and analysis files were stored in password‐protected digital folders accessible only to the research team. Identifiable information was removed during transcription, and the research data will be retained securely for 5 years after publication and then permanently deleted. The anonymized transcripts were then imported into MAXQDA Analytics Pro 2020 to facilitate systematic data management and coding. The analysis was conducted using the original Turkish transcripts. Quotations selected for publication were translated into English and checked by bilingual members of the research team for semantic equivalence.

### 2.7 Data Analysis

The data were analyzed using Colaizzi's descriptive phenomenological method (Colaizzi 1978). First, all transcripts were read repeatedly to gain an overall sense of participants' accounts. Second, significant statements directly related to the phenomenon under investigation were identified. Third, formulated meanings were developed from these significant statements. Fourth, related meanings were grouped into clusters and organized into themes that reflected shared aspects of the participants' lived experiences. Fifth, an exhaustive description of the phenomenon was developed based on the thematic structure. Sixth, the fundamental structure of the experience was refined through iterative team‐based analytic discussions. Analysis was led by the first author and discussed with the second and third authors to enhance analytic depth, consistency, and reflexive interpretation. Illustrative quotations were selected to support the themes and to preserve participants' experiential meanings. Finally, the emerging structure of the findings was returned to participants for confirmation of consistency with their experiences.

### 2.8 Rigor and Trustworthiness

Rigor was ensured in accordance with Lincoln and Guba's criteria of credibility, transferability, dependability, and confirmability (Lincoln and Guba 1985). The reporting of researcher characteristics, reflexivity, study context, data collection, analysis, and supporting quotations was guided by the Consolidated Criteria for Reporting Qualitative Research (COREQ) recommendations (Tong et al. 2007).

The research team consisted of three female academics holding PhD degrees in pediatric nursing, all of whom had experience in qualitative research and neonatal nursing. The interviews were conducted by the second researcher. The first and third researchers contributed to the study design, analytic discussions, and interpretation of the findings. No personal relationship existed between the interviewer and the participants before recruitment. However, the interviewer was living in an earthquake‐affected region, which enhanced her contextual awareness of the topic. Recognizing that this proximity might influence data collection and interpretation, the research team engaged in ongoing reflexive discussions throughout the study and critically considered how their professional backgrounds, assumptions, and prior interests in disaster preparedness might shape the analytic process.

Credibility was supported through in‐depth interviews, iterative team‐based discussions during analysis, participant checking of the emerging findings structure, and the use of direct quotations to reflect participants' experiences. Transferability was enhanced by providing detailed descriptions of the study context, participant characteristics, sampling strategy, data collection procedures, and analytic process. Although exact replicability is not the primary aim of qualitative phenomenological research, dependability was strengthened by transparently documenting the methodological procedures, maintaining an analytic decision trail, and applying Colaizzi's descriptive phenomenological analysis systematically across the dataset. Dependability was also supported through the consistent use of the semistructured interview guide. Confirmability was supported through collaborative review of codes and themes, ongoing reflexive consideration of the researchers' assumptions and positions during data collection and analysis, and the preservation of anonymized transcripts, coding records, and analytic notes as part of the audit trail (Nowell et al. 2017).

### 2.9 Ethical Considerations

Ethical approval for this study was obtained from the Kocaeli University Non‐Interventional Clinical Research Ethics Committee, an institutional university ethics committee authorized to review noninterventional health research, in July 2025 (Approval No.: 2025/367). All procedures were conducted in accordance with the Declaration of Helsinki. Written and verbal informed consent was obtained from all participants prior to data collection. Confidentiality was maintained through anonymization of the transcripts and secure storage of the study data. Participants were informed that participation was voluntary and that they could decline to answer any question, pause or stop the interview, or withdraw from the study at any time without providing a reason.

## 3 Result

### 3.1 Participant Characteristics

A total of 21 nurses participated in the study. All participants were female and held a university degree. The mean age was 30.77 ± 4.09 years, ranging from 23 to 37 years. Most participants were married (66.6%, *n* = 14). In terms of professional experience, 61.1% (*n* = 13) had 10 years or more of overall nursing experience, and 66.6% (*n* = 14) had at least 5 years of experience in the NICU. NICU experience ranged from 8 months to 18 years, indicating variation in clinical seniority. Regarding educational background, 71.4% (*n* = 15) held a bachelor's degree and 28.6% (*n* = 6) had a master's degree (Table 2).

**TABLE 2:** Demographics of the participants.

Participant code	Gender	Age	Marital status	Working experience (year)	NICU experience	Education level
P1	Female	31	Married	5–9	10 months	MSc
P2	Female	25	Single	5–9	3 years	Bachelor's degree
P3	Female	29	Married	5–9	3 years	MSc
P4	Female	34	Married	15–20	10 years	Bachelor's degree
P5	Female	35	Single	15–20	9 years	Bachelor's degree
P6	Female	37	Single	15–20	18 years	Bachelor's degree
P7	Female	32	Married	15–20	14 years	Bachelor's degree
P8	Female	35	Married	5–9	3 years	Bachelor's degree
P9	Female	25	Married	5–9	3 years	Bachelor's degree
P10	Female	31	Married	10–14	13 years	Bachelor's degree
P11	Female	37	Married	15–20	9 years	MSc
P12	Female	32	Married	10–14	10 years	Bachelor's degree
P13	Female	28	Single	5–9	3 years	Bachelor's degree
P14	Female	36	Married	10–14	11 years	Bachelor's degree
P15	Female	27	Married	5–9	8 months	Bachelor's degree
P16	Female	36	Married	15–20	8 years	Bachelor's degree
P17	Female	35	Married	15–20	12 years	MSc
P18	Female	27	Married	5–9	4 years	MSc
P19	Female	23	Single	1–4	3 years	Bachelor's degree
P20	Female	27	Single	1–4	4 years	MSc
P21	Female	32	Married	10–14	5 years	Bachelor's degree

### 3.2 Themes

Three interrelated themes were identified from the participants' accounts: (1) Coordination and Communication Amid Chaos, (2) Infrastructure Disruption and Technology‐Dependent Neonatal Care, and (3) The Gap Between Disaster Knowledge and Practice. Together, these themes reflected how disaster response in NICUs was experienced not only as a clinical emergency but also as a disruption of routine systems that required nurses to improvise coordination, protect fragile infants under compromised conditions, and act in the absence of context‐specific preparedness (Table 3).

**TABLE 3:** Main themes and subthemes.

Main themes	Subthemes
Coordination and communication amid chaos	Breakdown of formal communication systems
	Improvised role negotiation and decision‐making
	Sustaining teamwork through instinctive and nonverbal collaboration
Infrastructure disruption and technology‐dependent neonatal care	Fragility of life‐sustaining equipment and systems
	Uncertainty and risk in neonatal evacuation
	Shortages and adaptation in essential supplies and ongoing care
The gap between disaster knowledge and practice	Limits of theoretical disaster training
	Absence of NICU‐specific practical preparedness

#### 3.2.1 Coordination and Communication Amid Chaos

Participants described the early phase of the earthquake response as a period of chaos, uncertainty, and urgency in which routine coordination systems were disrupted. Communication systems were often nonfunctional, roles were unclear, and formal disaster plans could not be immediately implemented. Under these conditions, nurses reported that care had to be sustained through rapid decisions, improvised communication, and shared responsibility among team members.

Several participants described how the loss of telephones, announcement systems, and other communication channels affected coordination within and between units. As one nurse explained,

Initially, there was complete chaos. Phones weren't working, the announcement system was down. We could only communicate by shouting or running to colleagues in nearby rooms. Everyone assumed responsibility, but since there was no pre‐established plan, we had to make on‐the‐spot decisions. (P1)

Similarly, another participant stated,

During the earthquake, we acted on instinct. Details such as which unit would do what and where the infants would be evacuated were determined on the spot. Coordination was weak. (P4)

Participants also described using improvised forms of interaction when formal communication systems were unavailable. Eye contact, brief verbal commands, hand signals, and flexible role enactment became important ways of sustaining care. One participant described this as follows:

We proceeded more by making eye contact and trying to understand what each other was doing, rather than talking. The sounds, screams, alarms, darkness … everything was chaotic. (P10)

Another participant stated, “Without any formal organization, we relied entirely on instinctive team solidarity. No one said, ‘That's not my job.’ Nurses, doctors, and technicians all acted together” (P11).

Support from external teams was also described as important as the response unfolded, particularly when local staff were themselves affected by the earthquake or unable to remain on duty. As one participant stated,

Some colleagues had to leave to reach their families or had been affected themselves, so they could not return to duty. (…) Healthcare personnel came from different cities to support us. They were really helpful. (P10)

Participants' accounts indicated that, although formal coordination was disrupted in the first hours of the disaster, care continued through improvised teamwork, flexible role sharing, and interpersonal solidarity under extreme pressure.

#### 3.2.2 Infrastructure Disruption and Technology‐Dependent Neonatal Care

Participants described disaster response in the NICU as a struggle to sustain technology‐dependent neonatal care when infrastructure, equipment, and logistical systems were disrupted. Interruptions in electricity, oxygen delivery, monitoring systems, and evacuation planning affected the continuity of care for fragile infants who required respiratory support, thermal stability, feeding, medication management, and close observation.

Several participants emphasized that existing disaster plans did not adequately address the realities of neonatal intensive care. General hospital‐level preparedness was perceived as insufficient for managing device‐dependent infants, limited transport options, and unit‐specific care priorities. As one nurse stated,

Frankly, we were not adequately prepared. General disaster management training had been provided, but there was no comprehensive drill or practical preparation specifically for neonatal intensive care. Had we had a plan for how to act in an earthquake scenario, safely evacuate infants, or handle equipment shortages, perhaps the chaos would not have been so severe. (P6)

Another participant described the effects of equipment and system failures as follows:

Equipment failed, monitors shut down, and oxygen systems went offline. During those few minutes before the generator activated, it felt as if life stopped. We didn't know what to do because we had no preparation for such a situation. (P17)

Evacuation was described as one of the most uncertain and risky aspects of care. Participants reported that decisions about where to move infants, how to transport them, and which infants to prioritize had to be made during the crisis. The need to move infants who were connected to oxygen lines, monitors, incubators, or phototherapy devices made evacuation especially difficult. One participant explained,

Evacuation was completely uncertain. Where would we take them, how, which infant first, how to transport devices … there was no preparation. Some incubators were very difficult to move; oxygen lines and monitors were connected. Some infants were on oxygen support, some under phototherapy. So, moving them quickly wasn't easy. (P10)

Participants also described challenges related to essential supplies and continuity of basic neonatal care. Although some participants reported that routine materials were initially available, others referred to difficulties in accessing special solutions, preterm‐specific feeds, and adequate respiratory support equipment during the crisis. One participant noted that routine materials were available but emphasized the lack of NICU‐specific evacuation preparation:

We didn't face material shortages; everything was sufficient. We did not have a separate bedside emergency evacuation plan or practical disaster drill for neonates. Only general drills were conducted. (P14)

Another nurse described how care was maintained through immediate adaptation:

Infants with respiratory distress were supported with Ambu bags, wrapped in blankets to maintain body temperature. Medication doses were checked, and external support was requested for feeding. Our main priority was ensuring survival and minimizing infection risk. (P16)

Participants' accounts showed that neonatal care during the earthquake required continuous adaptation to unstable infrastructure and limited logistical support. Nurses worked to maintain respiration, warmth, monitoring, feeding, medication safety, and infection control while the technological and material conditions of care were disrupted.

#### 3.2.3 The Gap Between Disaster Knowledge and Practice

Participants described disaster preparedness as a gap between formal disaster knowledge and what could be applied in the NICU during a real earthquake. Although some participants had received general disaster education or had participated in hospital‐wide drills, these were often described as insufficient, outdated, or too general for the specific needs of neonatal intensive care. Participants reported that they lacked practical guidance for transporting neonates, prioritizing equipment, allocating roles, and maintaining care under crisis conditions.

Several participants stated that previous disaster education had not prepared them for the realities of neonatal intensive care during an earthquake. As one nurse explained,

No, we hadn't attended such training. Honestly, we weren't prepared for a disaster scenario. General hospital drills had been conducted, but I don't recall any specific NICU drill. A few years ago, we received some disaster training, but it had been a long time. It needed updating. It was insufficient, especially because no specific NICU planning had been done. (P1)

Another participant described the difficulty of applying existing disaster plans during the crisis:

We had a disaster plan, but we were not fully prepared to implement it. Our routine stock of equipment was sufficient. Oxygen systems remained functional thanks to the generator. However, we were unprepared for a prolonged crisis. (P7)

Participants also emphasized the absence of NICU‐specific preparation for role allocation, equipment prioritization, and neonatal transport. One participant stated,

Critical details, such as how to transport neonates, which equipment to prioritize, and who would take on which role, were lacking. As a nurse in intensive care, I was torn between responsibility for the infants and my own child's safety. I wanted to know what to do, but we had no systematic preparation. (P9)

Similarly, another nurse said,

Honestly, I did not have clear, field‐adapted knowledge about what a neonatal nurse should do in a disaster. This topic was briefly mentioned during training but never practiced. Disaster plans, task allocations, and evacuation procedures were only on paper. When faced with reality, most of what we knew theoretically was useless. (P18)

Participants reported that the lack of regular, practical, and NICU‐specific training increased uncertainty during the early phase of the earthquake response. They described relying on instinct, previous clinical experience, and moment‐to‐moment judgment in situations where clearer preparation was needed. As one participant stated,

There was neither a drill nor instruction on what to do in such a situation. We were left with only a few pieces of information from books. (P17)

Participants' accounts indicated that disaster preparedness in the NICU required more than general theoretical knowledge. They emphasized the need for repeated, practical, and unit‐specific preparation that reflects the realities of neonatal care during disasters.

## 4 Discussion

This study explored how NICU nurses experienced earthquake response during the 2023 Türkiye–Syria earthquakes. The findings showed that disaster response in neonatal intensive care was experienced not only as a clinical emergency but also as a disruption of the organizational, material, and practical conditions that normally sustain safe neonatal care. Nurses described difficulties in maintaining coordination and communication, preserving technology‐dependent care under unstable infrastructure, and translating general disaster knowledge into practical, unit‐specific action. These findings indicate that disaster preparedness in NICUs cannot be reduced to the existence of written plans or general hospital‐level training. Rather, it requires systems that enable coordinated team action, continuity of life‐sustaining technology, safe evacuation, and rehearsed neonatal care practices under crisis conditions.

A central contribution of this study is that it makes visible the distinctive vulnerability of neonatal intensive care during disasters. Previous literature has emphasized that NICUs are highly dependent on continuous electricity, oxygen delivery, monitoring, thermal regulation, and specialized equipment (Barfield and Krug 2017; Eskandar‐Afshari et al. 2020; Ma et al. 2020). The present findings extend this literature by showing how these dependencies were experienced by nurses during the early phase of a real earthquake response. For participants, infrastructure disruption was not an abstract organizational problem; it directly affected the possibility of maintaining respiration, monitoring, warmth, medication safety, feeding, and infection control for fragile infants. This highlights the need to understand NICU disaster response as a form of highly technical and time‐sensitive care in which system failures can rapidly become clinical risks.

The findings also show that communication and coordination are central to neonatal disaster response. Participants described the early hours of the earthquake as chaotic, with nonfunctioning phones, disrupted announcement systems, unclear roles, and decisions made in the moment. Similar challenges have been reported in disaster nursing and critical care preparedness studies, where communication breakdowns, weak leadership structures, and unclear role allocation can undermine effective response (Almukhlifi et al. 2021; Annak Mercan et al. 2025; Sellers et al. 2022). In this study, however, the consequences of disrupted coordination were intensified by the simultaneous needs of multiple fragile infants. When communication systems failed, nurses had to rely on eye contact, brief verbal commands, hand signals, and informal role sharing. These improvised strategies helped sustain care, but they also revealed the absence of a fully operational, unit‐specific response structure.

This distinction is important because the ability of nurses to improvise should not be interpreted as evidence of adequate preparedness. Rather, it shows how frontline staff compensated for organizational gaps through professional commitment, flexibility, and team solidarity. Similar forms of adaptive teamwork have been described in disaster contexts, particularly when formal systems are delayed or overwhelmed (Annak Mercan et al. 2025; Topal et al. 2024). Nevertheless, reliance on improvisation may increase uncertainty and place additional emotional and professional burden on nurses. In NICUs, where decisions about oxygen, warmth, monitoring, transport, and prioritization may need to be made within minutes, preparedness must support rapid shared situational awareness, predefined responsibilities, and reliable backup communication pathways.

Evacuation emerged as another important area requiring deeper attention. The findings suggest that neonatal evacuation cannot be understood as a simple movement of patients from one location to another. Instead, it involves the simultaneous management of clinical risk, equipment dependency, thermal protection, respiratory support, infection prevention, and prioritization. This is consistent with literature emphasizing the complexity of evacuating vulnerable and critical pediatric or neonatal patients during disasters (Mousavipour and Sohrabizadeh 2021; Thomas et al. 2020; Van Stralen et al. 2021). In the present study, participants' accounts of infants connected to oxygen lines, monitors, incubators, or phototherapy devices demonstrate how evacuation planning must be adapted to the physiological fragility and technological dependency of neonates. General evacuation plans may therefore be insufficient unless they include bedside‐level neonatal scenarios, equipment prioritization, staff role allocation, and transport procedures.

Another important finding concerns the gap between formal disaster knowledge and practice. Participants did not describe preparedness as a complete absence of information; rather, they described a mismatch between general disaster education and the realities of NICU care during an earthquake. Written plans and general drills were perceived as insufficient when nurses faced questions such as how to transport neonates, which equipment to prioritize, who would assume specific roles, and how care would continue during prolonged disruption. This finding is consistent with studies showing that disaster preparedness is weakened when education remains theoretical and is not reinforced through regular, scenario‐based, and unit‐specific drills (Becker et al. 2021; Kuppanda et al. 2021; Sellers et al. 2022). For NICU nurses, preparedness must therefore be embodied through repeated practice rather than assumed through policy documents alone.

The findings also point to the emotional and relational dimensions of NICU disaster response, although these were not the primary focus of the present study. Some participants referred to the tension between responsibility for fragile infants and concern for their own families or colleagues. This reflects the dual position of nurses as both healthcare professionals and disaster‐affected individuals. During large‐scale disasters, nurses may be expected to sustain care while simultaneously experiencing personal fear, uncertainty, and concern for loved ones. Although the interview guide primarily focused on clinical disaster response, coordination, infrastructure, evacuation, and preparedness, the emotional burden of NICU nurses and their communication with infants' families remains important areas for future research. These dimensions should also be considered in disaster planning through staff support, debriefing mechanisms, and family communication protocols.

Taken together, the findings suggest that NICU disaster preparedness should be understood as a unit‐specific, practice‐based, and systems‐oriented process. Preparedness efforts should include reliable backup systems for electricity, oxygen, monitoring, and essential supplies; clear communication pathways; predefined team roles; bedside‐level evacuation plans; and regular simulation‐based drills tailored to neonatal care. Training should not only address general disaster principles but also include realistic NICU scenarios involving equipment failure, transport of device‐dependent infants, thermal protection, prioritization, documentation, and family communication. Such approaches may help reduce uncertainty, strengthen nurses' confidence, and support safer neonatal care during future disasters.

This study contributes to the disaster nursing and neonatal care literature by providing qualitative insight into how NICU nurses experienced the early phase of earthquake response in a highly vulnerable and technology‐dependent care environment. The findings highlight that the safety of neonatal care during disasters depends not only on individual nurses' competence and commitment but also on the preparedness of the organizational, infrastructural, and educational systems that support their work. Strengthening these systems is essential for building disaster‐resilient neonatal intensive care services.

## 5 Limitations

This study has several limitations. First, the findings are based on retrospective accounts, and participants' recollections may have been influenced by the passage of time and the emotional intensity of the earthquake experience. Second, data were collected through online interviews, which may have limited the observation of nonverbal cues and the depth of interaction in some interviews. Third, the study reflects only the perspectives of NICU nurses; the views of physicians, managers, technical staff, or family members were not included. In addition, although some participants briefly referred to personal and family‐related concerns, the interview guide primarily focused on clinical disaster response, coordination, infrastructure disruption, evacuation, and preparedness. Therefore, nurses' emotional experiences and their communication with infants' families were not explored in depth. Future studies should specifically examine the emotional burden of NICU nurses and family communication during neonatal disaster response. Finally, as a qualitative study conducted in a specific disaster context, the findings are intended to offer contextual depth and transferability rather than statistical generalizability.

## 6 Conclusion

This study showed that disaster response in neonatal intensive care was experienced not only as a clinical emergency, but also as a disruption of the organizational, material, and practical conditions that sustain safe care. NICU nurses described major challenges in maintaining coordinated action, preserving technology‐dependent care under unstable infrastructure, and responding in the context of preparedness that often remained theoretical rather than actionable. These findings highlight the need for NICU‐specific disaster preparedness that goes beyond general hospital planning and includes team‐based coordination, infrastructure‐sensitive care planning, and regular practical training tailored to neonatal care. Strengthening these areas may help reduce uncertainty and support safer, more effective disaster response in neonatal intensive care settings.

### 6.1 Relevance for Clinical Practice

The findings of this study have direct relevance for clinical practice in neonatal intensive care settings. They show that disaster preparedness in the NICU must go beyond general hospital‐level planning and address the specific clinical and organizational demands of technology‐dependent neonatal care. In practice, this requires clearly defined team roles, reliable communication pathways, evacuation procedures tailored to fragile infants, and contingency planning for interruptions in electricity, oxygen supply, monitoring, and essential materials. The findings also highlight the importance of regular, simulation‐based, NICU‐specific disaster training to strengthen coordination, reduce uncertainty, and support timely decision‐making under pressure. Incorporating these elements into routine preparedness efforts may enhance both staff readiness and the safety of neonatal care during future disasters.

## Author Contributions

**Birgül Erdoğan:** conceptualization, methodology, formal analysis, writing – original draft, visualization, investigation, validation. **Sümeyra Topal:** methodology, data curation, writing – review and editing, software, resources, project administration. **Sinem Yalnızoğlu Çaka:** conceptualization, methodology, writing – review and editing, supervision, funding acquisition.

## Funding

The authors have nothing to report.

## Ethics Statement

Ethical approval for this study was obtained from the Kocaeli University Non‐Interventional Clinical Research Ethics Committee, an institutional university ethics committee authorized to review noninterventional health research, in July 2025 (Approval No.: 2025/367). All procedures were conducted in accordance with the Declaration of Helsinki. Confidentiality was maintained through anonymization of the transcripts and secure storage of the study data. Participants were informed that participation was voluntary and that they could decline to answer any question, pause or stop the interview, or withdraw from the study at any time without providing a reason.

## Consent

Written and verbal informed consent was obtained from all participants who agreed to take part in the study.

## Conflicts of Interest

The authors declare no conflicts of interest.

## Supporting information


**Data S1:** COREQ checklist.

## Data Availability

The data that support the findings of this study are available on request from the corresponding author. The data are not publicly available due to privacy or ethical restrictions.
