# Supplementary material for: Coordinating Earthquake Response in Neonatal Intensive Care: A Phenomenological Exploration of Nurses' Experiences
Source: Nurs Health Sci. 2026 Jul 18;28(3):e70394. doi: 10.1111/nhs.70394 (PMC13379728; doi:10.1111/nhs.70394)
Supplement: Supplementary file 1 — Data S1: COREQ checklist. [file NHS-28-e70394-s001.docx]

**COREQ (Consolidated Criteria for Reporting Qualitative Research) Checklist**

| Item | Description |
| --- | --- |
| Domain 1: Research Team and Reflexivity | |
| 1. Interviewer/facilitator | The interviews were conducted by the second author. |
| 2. Credentials | All three authors hold PhD degrees in pediatric nursing. |
| 3. Occupation | The researchers are female academics in pediatric nursing. |
| 4. Gender | Female. |
| 5. Experience and training | All authors had prior experience in qualitative research and neonatal nursing. The research team also had prior academic interest in disaster preparedness and qualitative health research. |
| 6. Relationship established | No personal relationship existed between the interviewer and the participants before recruitment. Initial contact was established through professional networks using snowball sampling. |
| 7. Participant knowledge of the interviewer | Participants were aware that the interviewer was a researcher in pediatric nursing and that the study focused on disaster response experiences in neonatal intensive care. |
| 8. Interviewer characteristics | The interviewer was living in an earthquake-affected region, which provided contextual familiarity with the topic. The research team engaged in ongoing reflexive discussions to consider how their professional backgrounds, assumptions, and contextual positions might influence data collection and interpretation. |
| Domain 2: Study Design | |
| 9. Methodological orientation | Qualitative phenomenological approach; data were analysed using Colaizzi’s descriptive phenomenological method. |
| 10. Sampling | Snowball sampling of neonatal intensive care unit nurses from 11 earthquake-affected provinces. |
| 11. Method of approach | Participants were contacted through professional networks, and interviews were conducted online via Zoom. |
| 12. Sample size | 21 nurses participated in the study. |
| 13. Non-participation | No refusals or withdrawals were reported. |
| 14. Setting of data collection | Data were collected through online interviews via Zoom. |
| 15. Presence of non-participants | Only the interviewer and participant were present during the interviews. |
| 16. Description of sample | Participant characteristics included age, marital status, education, overall professional experience, and neonatal intensive care unit experience. |
| 17. Interview guide | A semi-structured interview guide was developed based on the literature and the research team’s expertise, and was refined through feedback from five experts. |
| 18. Repeat interviews | No repeat interviews were conducted. |
| 19. Audio/visual recording | Interviews were audio-recorded with participant consent. |
| 20. Field notes | No formal field notes were reported. |
| 21. Duration | Interviews lasted approximately 45–60 minutes. |
| 22. Data saturation | Recruitment continued until sufficient depth and richness of experiential accounts had been achieved and no substantially new meanings relevant to the study aim were emerging during the analytic process. |
| 23. Transcripts returned | The emerging structure of the findings was returned to participants for confirmation of consistency with their experiences. |
| Domain 3: Analysis and Findings | |
| 24. Number of data coders | Analysis was led by the first author and discussed with the second and third authors through team-based analytic discussions. |
| 25. Description of coding tree | The main themes and subthemes are presented in Table 3. |
| 26. Derivation of themes | Themes were derived inductively from the data using Colaizzi’s descriptive phenomenological method. |
| 27. Software | MAXQDA Analytics Pro 2020 was used for data management and coding. |
| 28. Participant checking | Yes. The emerging structure of the findings was returned to participants for confirmation. |
| 29. Quotations presented | Participant quotations (P1, P2, etc.) were presented to support the findings. |
| 30. Data and findings consistent | Findings were grounded in participant accounts and supported by direct quotations. |
| 31. Clarity of major themes | Three major themes were clearly presented: struggling to sustain coordinated action in the midst of chaos; providing technology-dependent neonatal care under disrupted infrastructure; and feeling unprepared: when disaster knowledge remained on paper. |
| 32. Clarity of minor themes | Related subthemes were presented in Table 3. |
